# Effectiveness of Physical Activity Support Combined With Continuous Glucose Monitoring by a Physical Therapist in Preconception Care for a Woman With Type 2 Diabetes Mellitus: A Case Study

**DOI:** 10.7759/cureus.81544

**Published:** 2025-03-31

**Authors:** Kengo Taketani, Takuo Nomura, Tetsuji Okawa

**Affiliations:** 1 Department of Rehabilitation, Toyota Memorial Hospital, Toyota, JPN; 2 Department of Rehabilitation, Kansai Medical University, Hirakata, JPN; 3 Department of Endocrinology and Diabetes, Toyota Memorial Hospital, Toyota, JPN

**Keywords:** continuous glucose monitoring, physical activity support, physical therapist, preconception care, type 2 diabetes mellitus

## Abstract

Diabetes mellitus represents a major public health challenge worldwide, with type 2 diabetes mellitus (T2DM) accounting for the majority of cases. Urbanization and lifestyle changes are reportedly contributing to the increasing incidence of T2DM worldwide. The prevalence of T2DM is also increasing among women who wish to become pregnant, owing to the growing overall proportion of women with T2DM, the increasing prevalence of obesity, and the rising average age of childbirth. Preconception care is warranted in this demographic to optimize glycemic control, improve pregnancy outcomes, and reduce the risk of congenital anomalies and perinatal complications. Educational support that includes not only glycemic control but also increased physical activity (PA) and lifestyle modifications is important to delivering effective preconception care.

Herein, we report the case of a woman in her 30s with T2DM, hypertension, and dyslipidemia. Her early glycemic control was suboptimal (glycated hemoglobin: 8.9%, time in range (TIR): 36.6%), her pregnancy preparation was delayed, and a personalized PA program was eventually introduced that included continuous glucose monitoring (CGM) reviewed by a physical therapist. The intervention lasted four months and included continuous feedback and adjustments to the timing, intensity, and activity goals of the patient’s exercise regimen based on her CGM trends. A specific PA target of 8,000-10,000 steps per day was established to promote increased daily movement. The intervention also incorporated a combination of aerobic exercise (walking) and resistance training tailored to the patient’s condition and lifestyle. This intervention led to improvements in her blood glucose markers, treatment satisfaction related to diabetes, health-related quality of life, and independence.

The patient’s TIR increased from 36.6% to 77%, and her PA increased from 2500 to 9500 steps/day. This case study highlights the potential of CGM to promote real-time feedback and behavior modification in patients with T2DM, particularly those attempting pregnancy. PA support combined with CGM can effectively manage blood glucose levels, increase motivation, and improve overall health in ways that are highly beneficial to integrate into preconception care regimens.

This study emphasizes that PA support combined with CGM is effective for increasing glycemic control and PA levels, thus improving lifestyle habits and preparing women with T2DM for pregnancy. We advocate for the wider adoption of PA support interventions combined with CGM by physical therapists as a standard practice in preconception care and emphasize the role of this approach in terms of improving long-term metabolic health prior to conception. Further research is warranted to validate these findings and optimize intervention protocols.

## Introduction

Diabetes is a critical public health issue in Japan. According to the International Diabetes Federation’s Diabetes Atlas, the estimated number of adults aged between 20 and 79 years living with diabetes was approximately 11 million in the country in 2021. Japan’s prevalence of prediabetes is also notably high, suggesting that over 20 million people in the country may be affected by diabetes or prediabetes in some form [1].

Type 2 diabetes mellitus (T2DM) accounts for the majority of these cases, and it has become clear that urbanization and lifestyle changes have been increasing its incidence worldwide [2]. As part of this overall rise, the proportion of women with T2DM in Japan has been reported to be growing, and the number of pregnancies among women with T2DM has also risen. This trend can be attributed to increases in the prevalence of obesity and the median age of pregnancy in the country [3].

International surveys have confirmed an increase in pregnancies among women with T2DM worldwide, particularly in developed countries such as Japan. Societal aging and the increasing prevalence of obesity have also been noted as significant factors contributing to this reality [4].

Pregnancy with T2DM is associated with an increased rate of preterm delivery and significant impacts on fetal health, posing risks to both the mother and infant [5]. If a woman with T2DM wishes to have a baby, appropriate glycemic control before pregnancy is particularly important in order to prevent malformations in the baby and the exacerbation of diabetic complications in the mother.

Pregnancy outcomes are influenced not only by glycemic control, but also by a range of other factors such as diabetes-related complications, comorbidities, weight control, and physical activity (PA) levels. PA plays a crucial role in preconception care, as it has been shown to improve metabolic health, enhance insulin sensitivity, and support weight management - all of which contribute to better pregnancy outcomes. The American College of Obstetricians and Gynecologists (ACOG) recommends that women engage in ≥150 min of moderate-intensity aerobic exercise per week, which can be achieved by performing 30 min of exercise five days per week [6]. Furthermore, resistance training is encouraged to improve muscle strength, support glycemic control, and reduce insulin resistance during both preconception and pregnancy. Women who were physically active prior to conception can safely continue their habitual high-intensity aerobic activities during pregnancy, as long as no contraindications exist [6]. These guidelines underscore the importance of incorporating structured PA into preconception care to optimize maternal health, enhance blood glucose control, and reduce the risks of gestational complications.

Preconception care, including exercise-related education, can improve glycated hemoglobin (HbA1c) levels during the early stages of pregnancy. This can, in turn, reduce the risks of birth defects, miscarriage, perinatal death, preterm delivery, and small-for-gestational-age infants (i.e., newborns with birth weights under the 10th percentile for their gestational age) later on [7]. Given these benefits, structured PA programs should be integrated into standard preconception care for women with T2DM to improve both maternal and fetal outcomes.

Efforts should be made to promote a better understanding of the importance of planned pregnancy, including effective contraception. According to the American Diabetes Association, a target HbA1c level of <6.0% is considered optimal, with <6.5% being an acceptable goal for most women planning pregnancy. However, to avoid severe hypoglycemia, a more relaxed target of <7.0% may be appropriate for some patients [8].

A multifaceted approach to pregnancy preparation is recommended in women with T2DM, with exercise regimens both before and during pregnancy representing an important pillar of glycemic control and the prevention of complications. Exercise therapy improves insulin sensitivity and reduces glycemic variability [9]. A growing number of studies have evaluated the efficacy of exercise therapy in patients with T2DM who wish to have children and have shown that appropriate exercise therapy may contribute to improved blood glucose and insulin sensitivity, thereby improving pregnancy outcomes [10]. Exercise before and during pregnancy has also been shown to help manage blood glucose levels and reduce birth-related complications [11].

In recent years, continuous glucose monitoring (CGM) has attracted increasing attention and gained popularity as a new tool for managing diabetes. CGM facilitates real-time monitoring of blood glucose fluctuations during daily life and exercise, as well as the visualization of the effects of lifestyle habits such as exercise and diet on blood glucose levels. It is expected to represent an effective means of promoting behavioral changes in patients, as it provides more detailed information on blood glucose fluctuations than conventional intermittent self-monitoring of blood glucose (SMBG) [12].

CGM has been used in a range of clinical settings and has been introduced as a component of research projects and treatment regimens for patients with T2DM, as well as those with gestational and type 1 diabetes. It has been reported to be particularly effective for monitoring blood glucose during the acute phase of the disease, confirming the efficacy of exercise therapy, adjusting diet plans, and monitoring blood glucose fluctuations overnight.

Recent studies have confirmed that CGM is effective for improving HbA1c control and increasing time in range (TIR), which is defined as the time that the blood glucose levels of patients with diabetes remain within their target range. Martens et al. showed that the use of CGM in patients with T2DM significantly reduced HbA1c levels by 1.1% after eight months compared to conventional SMBG [13]. Allen et al. similarly showed that CGM use increased PA and decreased HbA1c levels by 1.2% in comparable patients [14].

The guidelines of the Japan Diabetes Society also recommend the use of CGM in patients with gestational diabetes and other forms of the disease who wish to become pregnant, where strict glycemic control is required. CGM use improves glycemic control and reduces the risk of complications in patients with diabetes who are pregnant or planning to become pregnant [15].

Personalized PA support combined with CGM is essential for maintaining both maternal and fetal health for women with T2DM who wish to have children. This case report describes a case wherein personalized PA support combined with CGM was successful in terms of improving glycemic control and preparing a patient with T2DM for pregnancy. The study also aimed to demonstrate how PA support delivered by a physical therapist and combined with CGM may represent an effective therapeutic approach for the future management of diabetes mellitus.

## Case presentation

The patient was a 34-year-old woman with T2DM who worked as a non-medical caregiver at a residential care facility. She had been diagnosed with diabetes in her teenage years and had been receiving treatment since then. Although her profession involved assisting others with daily living activities, she had limited knowledge of pharmacological treatment and exercise therapy options for diabetes management. The patient had not previously received any formal instruction on exercise therapy from a physiotherapist.

She wished to have a child and was undergoing Kaufmann therapy at our institution to regulate her hormonal balance and address ovulatory dysfunction. Kaufmann therapy, first introduced by Carl Kaufmann in 1932, is a hormone replacement therapy designed to induce menstruation in women with ovulatory dysfunction [16]. This therapy mimics the hormonal environment of a natural menstrual cycle through the sequential administration of exogenous estrogen and progestin, promoting both the proliferative and secretory phases of the endometrium and ultimately inducing withdrawal bleeding. This regimen typically involves administering estrogen from days 5 to 21 of the menstrual cycle, followed by the addition of progestin during the latter 12 days. Menstrual-like withdrawal bleeding usually occurs within two to seven days after completing the therapy. In addition to CGM, diet, and medication, an additional intervention was initiated by a physiotherapist to strengthen her preparation for pregnancy, as inadequate glycemic control had delayed her conception planning. The CGM used in this study was the FreeStyle Libre (Abbott Diabetes Care, Alameda, CA, USA).

The patient’s only specific diabetes-related complication was stage 1 nephropathy with no retinopathy or peripheral neuropathy. Her comorbidities included hypertension and dyslipidemia. Physical examination at her first visit revealed a height of 166 cm, weight of 91.3 kg, body mass index of 33.8 kg/m^2^, HbA1c glycemic control index of 8.9, mean random blood glucose level of 210.4 mg/dL, TIR of 36.6%, time above range (TAR) of 63.3%, time below range (TBR) of 0%, glycemic management indicator (GMI) of 8.3, area under the blood glucose level curve (AUC) of 131.9 mg-h/dL, and mean amplitude of glycemic excursions (MAGE) of 129.4.

The patient was treated via pharmacotherapy, with an insulin lispro dose of 45 U/day (Lyumjev, 100 U/mL) administered subcutaneously, divided into three doses before meals, as well as insulin glargine at 20 U/day (Lantus XR, 300 U/mL) delivered subcutaneously at bedtime (Lumjeb: 45 units/day and Lantus XR: 20 units/day).

**Figure 1:**
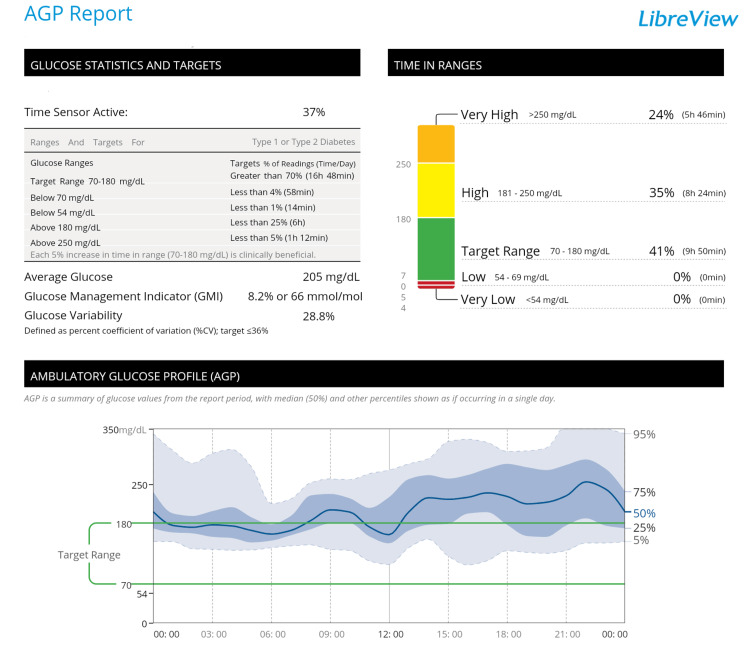
Ambulatory glucose profile (AGP) report. To enhance the clarity of the AGP graph, we have annotated the key elements as follows: The solid line (central thick line) represents the median (50th percentile) continuous glucose monitoring (CGM) data, indicating the central trend of blood glucose fluctuations throughout the day. The dark blue region corresponds to the interquartile range (IQR, 25th-75th percentile), representing the middle 50% of blood glucose values, providing insight into the typical variability around the median. The light blue region represents the 10th-90th percentile range, illustrating the broader spread of blood glucose fluctuations, excluding extreme hyperglycemic and hypoglycemic values. This allows for a visual representation of the overall glycemic variability while filtering out outliers.

Starting with the initial counseling session, the patient received five 30-minute physical support sessions with a physical therapist every month for four months. During these sessions, the therapist continuously assessed her progress, lifestyle, and blood glucose fluctuations as recorded via the CGM and systematically adjusted her treatment goals. Monthly feedback was provided, and the intervention plan was modified based on the patient's progress and challenges to set realistic and personalized goals for the type and amount of exercise targeted. The systematic timeline and goal-setting process are detailed in Table 1. The obtained CGM data (i.e., ambulatory glucose profile (AGP) report) are presented in Figure 1.

**Table 1:** Details regarding physical activity support interventions. CGM: continuous glucose monitoring; TIR: time in range (percentage of time wherein blood glucose levels fell within the target range); PA: physical activity; App: mobile application (used to remotely monitor the patient’s exercise status)

Intervention Number	Face-to-Face Frequency (Per Month)	Remote Frequency (Per Week)	Steps Target
First	Organize the relationship between blood glucose fluctuation and daily life based on past CGM data; Understand the duration of hyperglycemia and its impact on exercise therapy; Improve overall activity to counter the persistence of daily hyperglycemia.	Not applicable	3,000 steps
Second	Check exercise status and provide feedback based on CGM data; Confirm improvements in step count; Provide TIR feedback regarding the relationship between blood glucose variations and daily life.	App-based check of exercise status	4,000 steps
Third	Confirm PA levels and number of steps; Reassess blood glucose variations and adjust exercise recommendations.		5,000 steps
Fourth	Confirm PA levels and number of steps; Reset exercise load/volume according to level of achievement.		8,000 steps

The AGP report identified persistent daily hyperglycemia, particularly after lunch and dinner, as a daily issue for the patient. AGP is a standardized graphical representation of CGM data that allows for a more intuitive assessment of blood glucose variability and trends over time. Considering the patient’s low level of exercise during the day, a PA support program was also designed. Specifically, she was instructed to perform resistance training and aerobic exercise to mitigate her elevated blood glucose levels following lunch and dinner. The exercise prescription followed the frequency, intensity, time, and type (FITT) principle with a frequency of two to three times per week (ideally daily); intensity of moderate, guided by a perceived exertion level of 13 on the Borg scale; time of 10-30 min per session, performed after lunch and dinner; and type being a combination of walking and resistance training. This structured approach aimed to optimize glycemic control while ensuring adherence and safety.

A structured exercise regimen was proposed that she could implement with her clients at the facility where she worked as a caregiver. We also instructed the patient to walk and perform resistance training during the post-dinner period when her blood glucose levels rose postprandially. To mitigate the potential risks associated with exercise therapy, specific considerations were taken into account. These included the risk of hypoglycemia, for which the patient was advised to monitor her blood glucose levels before and after exercise and consume a small carbohydrate snack if necessary. Musculoskeletal strain and joint pain were also considered. Follow-up sessions were conducted to assess any discomfort, and the patient was instructed to temporarily suspend certain exercises if pain occurred. Improvements to non-exercise-related thermogenesis and overall PA levels were targeted by reducing prolonged sitting during periods of elevated blood glucose, particularly after lunch and dinner, as well as before bedtime, when her hyperglycemia tended to persist. The patient was instructed to incorporate "breaks" - brief interruptions in sedentary behavior - to enhance daily activity levels. Specifically, she was advised to stand up and engage in one to two minutes of stretching or light movement for every 30-60 mins of prolonged sitting (e.g., during desk work) to mitigate prolonged sedentary time and promote overall PA throughout the day.

As part of a multidisciplinary care approach, dietary recommendations were provided by a registered dietitian, while the physical therapist supplemented this guidance by explaining its relevance to PA and glycemic management. These recommendations were developed collaboratively, ensuring alignment with the patient’s overall treatment plan and facilitating their implementation at the facility where she worked as a caregiver.

The initial goal was set at approximately 3,000 steps/day, before being gradually increased according to the level of achievement. In the second and later sessions, we similarly assessed the relationship between the patient’s amount of PA, daily habits, and CGM data. A mobile application with a messaging feature was used to monitor her PA levels and provide real-time feedback, enabling continuous engagement between the patient and the healthcare team. Specific PA support details are shown in Table 1.

The intervention’s efficacy was determined by measuring the patient’s HbA1c, mean blood glucose level, TIR, TAR, TBR, GMI, AUC, and MAGE as indexes of glycemic control. Satisfaction with diabetes management, health-related quality of life, and self-efficacy were also assessed before and after the intervention.

Satisfaction with diabetes management was assessed using the Diabetes Treatment Satisfaction Questionnaire, while health-related quality of life was evaluated using the EuroQol 5-Dimensions 5-Level (EQ-5D-5L) survey. Self-efficacy was measured using the Enhanced Self-Efficacy Scale for Diabetes, the Self-Efficacy Scale for Diabetes, and the Self-Adjusted Self-Efficacy Scale. These evaluations were conducted by a physical therapist with over 15 years of clinical experience. All of the assessment tools used in this study have been validated in previous studies, wherein they demonstrated high internal consistency and established construct validity in terms of evaluating diabetes-related self-efficacy and quality of life.

**Figure 2:**
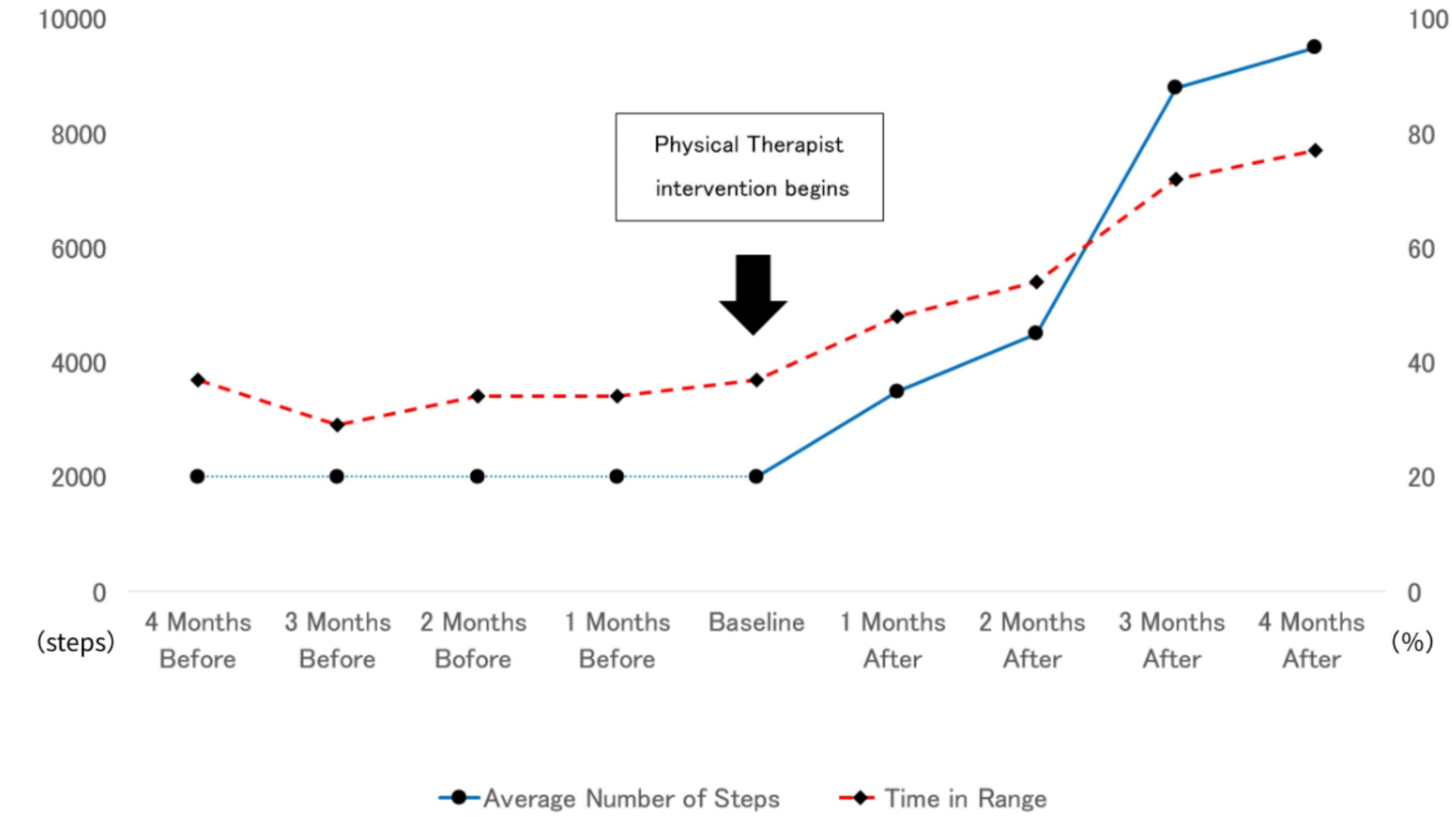
Relationship between average number of steps taken and time in range.

Although there were no major changes to the patient’s diet or medication, the intervention managed by the physical therapist resulted in improved glycemic control, satisfaction with diabetes management, health-related quality of life, and self-efficacy. These data are presented in further detail in Tables 2-3. The association between TIR (the main glycemic control index) and the average number of steps taken is shown in Figure 2.

**Table 2:** Changes in test value quantitative indicators at baseline and after the intervention. BMI: body mass index; HbA1c: glycated hemoglobin; HDL: high-density lipoprotein; LDL: low-density lipoprotein; TIR: time in range; TAR: time above range; TBR: time below range; GMI: glycemic management indicator; AUC: area under the blood glucose level curve; MAGE: mean amplitude of glycemic excursions

	Pre-intervention	Post-intervention	Normal Range	Unit
Weight	91.3	90.9	-	kg
BMI	33.8	32.7	18.5-24.9	kg/m²
Total cholesterol	232	201	140-199	mg/dL
Triglyceride	432	205	30-149	mg/dL
HDL cholesterol	43	43	40>	mg/dL
LDL cholesterol	140	128	60-119	mg/dL
LDL/HDL	3.3	3	>2	-
Blood glucose level (any time)	166	102	70<140	mg/dL
HbA1c	8.9	7	4.0-5.6	%
Mean blood glucose level	210.4	156.4	70-140	mg/dL
TIR	36.6	77	≥70	%
TAR	63.3	27	<30	%
TBR	0	0	<4	%
GMI	8.3	6.8	4.0-5.6	%
AUC	131.9	129.6	150-200	mmol/L·h
MAGE	129.4	96.7	<100	mg/dL
Average number of steps per day	2000	9500	-	steps/day

**Table 3:** Changes in treatment satisfaction and self-efficacy scores after the intervention. DTSQ: Diabetes Treatment Satisfaction Questionnaire; EQ-5D-5L: EuroQol-5 Dimension-5 Level; EQ-VAS: EuroQol Visual Analogue Scale; ESESD: Enhanced Self-Efficacy Scale for Diabetes; SESD: Self-Efficacy Scale for Diabetes

	Measure	Baseline	After the Intervention	Unit
Diabetes treatment satisfaction	DTSQ	9	30	points
Health-related quality of life	EQ-5D-5L	0.817	1	score
	EQ-VAS	30	80	score
Self-efficacy	ESESD	13	28	points
	SESD	8	18	points

The patient’s HbA1c decreased from 8.9% to 7.0% following the intervention, while her TIR increased from 36.6% to 72.2%. Her GMI also decreased from 8.3% to 6.8%, and her MAGE improved from 129.3 mg/dL to 96.6 mg/dL. Despite these metabolic improvements, significant weight loss was not observed. This may be attributed to potential increases in muscle mass induced by the structured PA program or the minimal changes made to the patient’s diet. However, as body composition measurements (e.g., fat and muscle mass) were not included in this study, further investigations are warranted to assess these hypotheses. This case study demonstrates that appropriate PA support through CGM enabled our patient to better understand the relationship between her blood glucose trends and PA levels, allowing her to appropriately adjust the timing and type of exercise she performed. The CGM's visual feedback of PA and glycemic control contributed to the patient’s increased motivation to exercise and alter her behavior. Her increased motivation to prepare for pregnancy also likely played a significant role in improving her glycemic control. Her indicators related to satisfaction with diabetes treatment, health-related quality of life, and self-efficacy also improved, suggesting that the intervention was associated with improvements in her satisfaction, self-efficacy, and PA level. It should be noted that the patient had also been using CGM prior to the intervention, which facilitated a continuous assessment of her blood glucose variations and trends.

## Discussion

Preconception lifestyle interventions administered as part of overall preconception care can lead to significant weight loss, improved metabolic health, and reduced risks of complications during pregnancy in women with T2DM who wish to have children [17]. Preconception care, including exercise-related education, has been reported to increase overall health knowledge and PA levels [18]. The present case report also demonstrates that PA support delivered by a physical therapist using CGM represents an effective way to improve PA levels and lifestyle habits in patients with T2DM who wish to have children.

PA support delivered by physical therapists is expected to gain increasing significance over the coming years, as the importance of preconception care must be fully explained and managed by a comprehensive team of specialists. In this study, the physical therapist's support plan was implemented as part of a multidisciplinary approach involving collaboration between physicians, nurses, and dietitians. The patient’s status and exercise therapy progress were regularly shared in real time through case conferences and electronic medical records. Data comparisons made before and after the CGM-supported PA intervention were able to promote behavioral changes in the patient that increased her self-efficacy, health-related quality of life, and motivation to exercise. Patients who are limited to conventional SMBG may find it difficult to understand their blood glucose fluctuations in detail as they adjust to the effects and timing of PA regimens.

CGM, on the other hand, facilitates the visualization of blood glucose fluctuations in real time and may, therefore, encourage patients to exercise based on the available data. In this study, the physiotherapist provided personalized exercise guidance tailored to the patient's CGM data, ensuring that the timing and intensity of exercise were adjusted according to her blood glucose levels.

Exercise support based on CGM data is particularly useful for visualizing the effects of exercise programs. This can help to motivate patients, increase their self-efficacy in terms of managing their blood glucose levels, and encourage sustained behavioral changes in their daily lives [19]. CGM is also useful as a tool to promote safe exercise when hypoglycemia and blood glucose fluctuations are anticipated, particularly in patients who wish to have children. It is important for such patients to avoid rapid blood glucose fluctuations and maintain stable glycemic control. PA support with CGM is important for preconception care and may promote lifestyle changes that persist even after delivery, thereby contributing to improved postpartum glycemic control and long-term health maintenance in patients with T2DM.

The intervention we administered in this study, which included cognitive behavioral strategies delivered by a practicing physical therapist, was associated with increased self-efficacy related to diabetes management - as demonstrated by a 35% increase in the patient’s PA, self-efficacy, and motivation [20]. However, it is important to acknowledge that the psychological scales were assessed by a single physical therapist, which may have introduced a potential bias due to emotional dependence between the evaluator and the patient.

The combination of CGM and PA support is important for improving both exercise and self-efficacy. This case study provides a valuable example of a practical application of CGM in preconception care. However, several scientific and clinical contexts help to highlight why exercise interventions are not currently implemented routinely in preconception care.

Healthcare providers face challenges in implementing such interventions, including a lack of research regarding the efficacy of exercise therapy interventions in the context of preconception care, as well as a lack of guidelines regarding the safety, optimal intensity, and frequency of exercise regimens. PA programs are not standardized, making it difficult to provide an appropriate exercise plan for each patient.

Many interventions administered by other professionals tend to focus more on pharmaceutical and dietary therapies, with PA support often being given a lower priority. On the patient side, many women who wish to become pregnant are excessively concerned about the effects of exercise on the fetus and may, therefore, choose to avoid it. Patients may also not be able to afford to start exercise routines because of time constraints, household chores, employment-related obligations, or other societal factors.

It is, therefore, important to accumulate further evidence concerning exercise support with CGM in the literature and standardize PA support programs by implementing them in more cases and clinical studies. It is important to promote active involvement in preconception care aimed at improving PA and lifestyle changes, controlling blood glucose, and reducing the risk factors associated with diabetes. In this context, the role of physical therapists specializing in exercise therapy is expected to become increasingly significant. Collaboration between diabetologists, obstetricians, gynecologists, and nutritionists may aid in the development of structured exercise programs to support optimal physical preparation for pregnancy. Additionally, personalized exercise therapy guided by CGM can facilitate stricter blood glucose control before conception, contributing to improved reproductive health outcomes for women with diabetes. There is a need to provide adequate education and support concerning appropriate PA at an earlier stage for patients with T2DM who wish to become pregnant.

## Conclusions

This case highlights the notion that personalized PA support with CGM as a component of preconception care can effectively promote behavioral changes, including increased self-efficacy, health-related quality of life, and motivation to exercise, ultimately leading to improved glycemic control in patients with T2DM. In terms of safety, no adverse events such as exercise-induced hypoglycemia, excessive hyperglycemia, or musculoskeletal injuries were observed during the intervention period. The patient was monitored regularly, and real-time CGM data allowed for immediate adjustments in exercise intensity and timing based on her glycemic trends. PA support combined with CGM extends beyond blood glucose monitoring, showing significant potential as a safe and effective approach for supporting exercise habits during preconception care.

The integration of CGM-based PA support by physical therapists is expected to become a new standard of care in the near future, contributing to improved glycemic control and quality of life in patients undergoing preconception care. Further research is warranted to validate these findings, analyze the safety and efficacy of this approach, and establish standardized intervention protocols tailored to preconception care in patients with T2DM.

## Appendices

Appendix A

Time in Range (TIR)

TIR is a metric derived from continuous glucose monitoring (CGM) that represents the proportion of time blood glucose levels remain within the target range (typically 70-180 mg/dL). This measure provides a more dynamic assessment of glycemic control, complementing traditional HbA1c values.

Time Above Range (TAR)

TAR represents the percentage of time during which blood glucose levels exceed the target range. It is used to assess the extent and risk of hyperglycemia, highlighting periods when blood sugar is too high.

Time Below Range (TBR)

TBR shows the percentage of time that blood glucose levels fall below the target range. This measure is crucial for monitoring and managing the risk of hypoglycemia, identifying times when blood sugar is too low.

Glycemic Management Indicator (GMI)

GMI is a calculated metric derived from the average blood glucose level that provides an estimate similar to HbA1c. It offers insights into long-term glycemic control based on daily blood glucose measurements.

Ambulatory Glucose Profile (AGP)

AGP is a standardized graphical format for representing CGM data, which provides a clearer visualization of blood glucose trends and variability over time.

Area Under the Curve (AUC)

AUC is a numerical integration of CGM-derived blood glucose data over time, providing a measure of total glucose exposure. It is commonly used to assess glucose variability, prolonged hyperglycemia, and overall glycemic burden. A higher AUC suggests greater fluctuations in blood glucose levels, which is critical for evaluating metabolic risk.

Mean Amplitude of Glycemic Excursions (MAGE)

MAGE measures the average amplitude of significant blood glucose fluctuations, capturing the extent of glycemic variability. It is an important metric for assessing the stability of blood sugar control.

Diabetes Treatment Satisfaction (Diabetes Treatment Satisfaction Questionnaire (DTSQ))

This indicates greater confidence in managing diabetes.

Health-Related Quality of Life (EuroQol 5-Dimensions 5-Level (EQ-5D-5L))

This reflects improved overall well-being.

Exercise Self-Efficacy (Enhanced Self-Efficacy Scale for Diabetes (ESESD))

This demonstrates increased confidence in sustaining an exercise routine.

Self-Directed Learning (Self-Efficacy Scale for Diabetes (SESD))

This shows enhanced self-management skills, including planning, execution, and problem-solving abilities.
